# Propolis: A natural biomaterial for dental and oral healthcare

**DOI:** 10.15171/joddd.2017.046

**Published:** 2017-12-13

**Authors:** Zohaib Khurshid, Mustafa Naseem, Muhammad S Zafar, Shariq Najeeb, Sana Zohaib

**Affiliations:** ^1^Department of Fixed Prosthodontics, College of Dentistry,King Faisal University, Hofuf, Saudi Arabia; ^2^Department of Preventive dental Sciences, College of Dentistry, Dar-Al-Uloom University, Riyadh, Saudi Arabia; ^3^Department of Restorative Dentistry, College of Dentistry, Taibah University, Madinah, Al Munawwarah, Saudi Arabia; ^4^Adjunct Faculty, Department of Dental Materials, Islamic International Dental College, Riphah International University, Islamabad, Pakistan; ^5^Private Dental Practitioner, Restorative Dental Sciences, Canada; ^6^Department of Biomedical Engineering, King Faisal University, Al-Hofuf, Saudi Arabia

**Keywords:** Antimicrobial, dentistry, natural biomaterials, oral health care, propolis, restorations

## Abstract

The field of health has always emphasised on the use of natural products for curing diseases. There are varieties of natural products (such as silk, herbal tea, chitosan) used today in the biomedical application in treating a large array of systemic diseases. The natural product "Propolis" is a non-toxic resinous material having beneficial properties such as antimicrobial, anticancer, antifungal, antiviral and anti-inflammatory; hence gain the attention of researchers for its potential for bio-dental applications. The study aims to explore the properties and chemistry of propolis concerning biomedical and dental applications. In addition, status and scope of propolis for current and potential future in bio-dental applications have been discussed. This review gives an insight to the reader about the possible use of propolis in modern-day dentistry.

## Introduction

**Figure 1:**
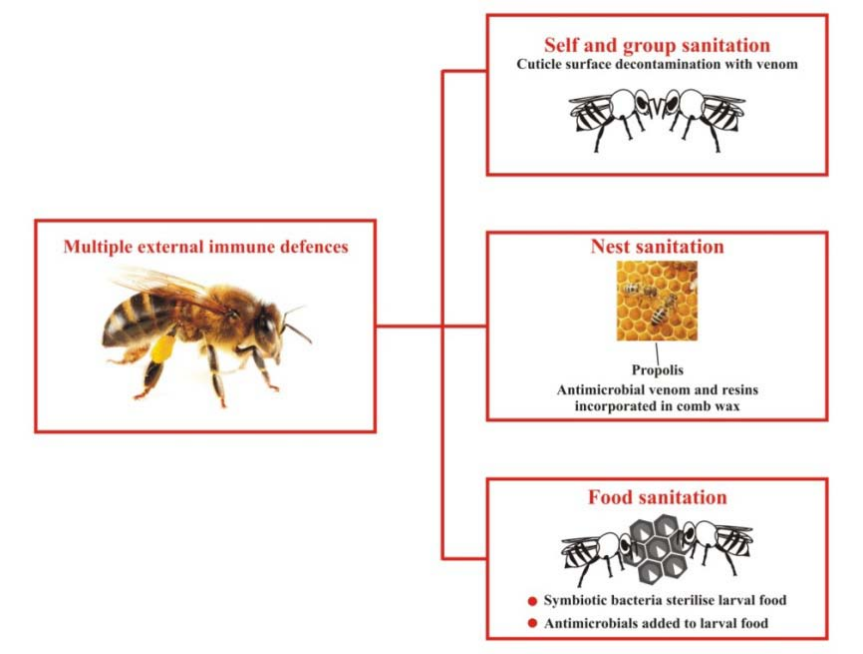
A schematic representation of possible associations of propolis and bees‏.

The field of health has always emphasized on the use of natural products for curing diseases rather than depending on the conventional allopathic medicine. There are varieties of natural products used today in the biomedical application in treating a large array of systemic diseases. These may include natural silk,^1,2^ chitosan,^3,4^ herbal tea^5^ and miswak.^6^ Propolis a non-toxic resinous natural substance exhibiting antimicrobial, anticancer, antifungal, antiviral and anti-inflammatory properties has gained attention in both the dental and medical fields. This wax-cum resin substance comes from Greek word “pro” (meaning outer wall) and “polis” (meaning city). This reflects the protective nature of the substance.^7,8^ Propolis is one of the natural substances made by bees for building and preservation of their hives. It kills pathogens, shields the honeycomb from rain and due to its adhesive nature prevents foreign guests from entering the hive (Figure 1).^7^

This naturally occurring substance has a wide range of overlooked benefits. It is categorized into twelve different types according to terrestrial location and physiochemical properties. However, only three different types of botanical origin have been identified.^8^ Propolis is considered the nucleus and powerhouse of nutrients.^9^ This resinous lipophilic material is sticky, soft and flexible when exposed to wheat but hard and breakable when cold.8 Propolis is primarily composed of resins (55‒60%). Waxes and fatty acids contribute around 30‒45% and aromatic oil and pollen about 10‒5%.^10^ Other substances may include minerals, vitamins and flavonoids. The biological activity of propolis is mostly linked with flavonoids and hydroxycinnamic acid.^11^ Research has revealed that it is difficult to standardize the chemical constituents and flavonoid contents of propolis as it is dependent on the environmental condition on the site of collection, on its origin and type of plant pollen and species of bees that produced it.^10,12^ Commercial availability of propolis is in the form of lozenges, topically applied cream, mouth rinses and toothpastes.^8^ The aim of this study is to explore the properties and chemistry of propolis concerning its biomedical and dental applications. In addition, status and scope of propolis for its contemporary and potential future bio-dental applications have been discussed‏.

### Chemical unpredictability of propolis

**Figure 2:**
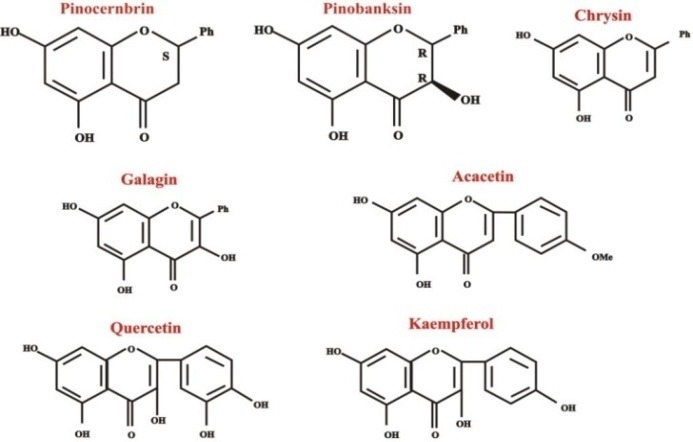
The nomenclature and chemical structures of various constituents of propolis‏.

The chemical variability of propolis is due to the different origin of plants, i.e. climatic and geographical whereabouts, flora at the site of collection and bee species.^12,13^ For the production of propolis, bees use secretions of different plants as well as substances discharged from wounds in plants, i.e. lipophilic materials on leaves, leaf buds, resins, gums and matrices.^14,15^ Therefore, there is a striking chemical variability in propolis composition mostly from the tropical regions. Kujumgiev et al^16^ compared the antibacterial, antiviral, antifungal and anti-inflammatory properties of propolis from different origins and concluded that all showed significant properties, including impotant antiviral properties. Similarly, Popova et al^17^ reported the same findings compared to the biological activity of propolis with geographical origin. The chemical constituents of propolis include chrysin, galangin, pinocembrin, pinobaskin found in a temperate climate. These are flavonoids without B-ring substituents. The major component of temperate propolis is caffeic acid phenethyl ester (CAPE).^14^ Similarly, the chemical composition of propolis originating from tropical regions includes prenylated phenylpropanoids (e.g., artepillin C), whereas propolis found in Pacific and African regions contains geranyl flavanones as the characteristic compounds (Figure 2).^14,18^

### Properties of propolis in medicinal care

**Figure 3:**
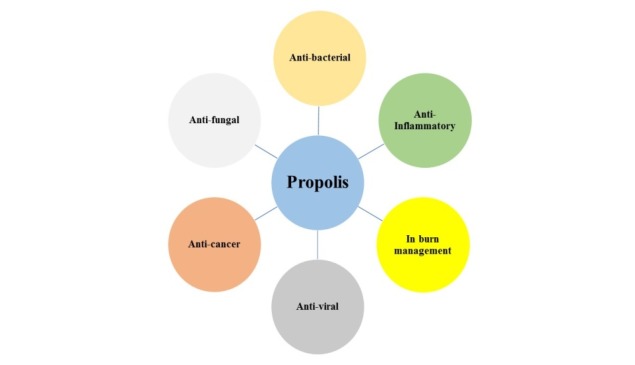
Useful properties and potential applications of propolis in the health care field‏.

Propolis, having a wide variety of therapeutic advantages, i.e. being cost-effective and biocompatible with the human cell, with no toxicity, limited allergic reaction and ready availability, can be used widely in medicinal care (Figure 3).^19,20^

### Antibacterial property of propolis

There is unequivocal evidence that propolis exhibits remarkable antibacterial properties despite modifications in chemical structures and collection from different geographical regions. Proof suggests that this natural resin is effective against gram-positive rods in addition to Mycobacterium tuberculosis, with restricted activity against gram-negative bacilli.^21^ The ethanolic extract of propolis (EEP) shows high efficacy against the strains of bacteroides and Peptostreptococcus but exhibits less efficiency against the strains of Clostridium, Eubacterium and Archnia.22 Three antimicrobial compounds were discovered from Brazilian propolis, mainly consisting of ^3,5^ diprenyl-4-hydroxycinnamic acid, 3-prenyl-4-dihdrocinnamoloxycinnamic acid and 22-dimethyl 6-carboxy-e-thenyl-2H-1-bezopyran, of which the initial compound shows the highest activity against bacteria and is one of the major antimicrobial compounds.^22,23^ Furthermore, EEP displayed synergism with certain antibiotics and demonstrated the capacity to improve the actions of antifungals. There is a growing medical interest in the antimicrobial potential of propolis alone or in combination with certain antibiotics and antifungals.^24^

### Antifungal action of propolis

Antifungals are used for the treatment and prevention of fungal infections. Commonly, these antifungal drugs are prescribed for the fungal infection of skin, hair, nail and oral candidiasis. Furthermore, they are used as a supportive therapy for patients suffering from denture stomatitis and added to denture tissue conditioners.^25,26^ Propolis extract shows excellent performance regarding in vitro tests against yeasts identified as onychomycosis agents. In low concentrations, propolis extract was not only found to be fungistatic but also fungicidal. C. tropicalis was found to be the most resilient whereas the Trichosporon species were the most vulnerable yeasts. The results reinforce the importance and the potential of propolis extract as a treatment for onychomycosis.^27^ The results of the study showed that all the yeasts tested were inhibited by low concentrations of propolis extract, including an isolate resistant to nystatin.^28^ Similarly, Ota et al studied antifungal activity of propolis extract on 80 different strains of Candida yeast and found the yeasts showed a clear antifungal activity with the following order of sensitivity: C. albicans>C. tropicalis>C. krusei>C. guilliermondii.^29^ Recently, Siquera et al assessed the fungistatic and fungicidal activity of propolis against different species of Candida using fluconazole as control. It was noted that propolis has fungistatic and fungicidal properties better than fluconazole.^30^

### Antiviral activity of propolis

Propolis extracts demonstrated high levels of antiviral activity against herpes simplex virus-1 (HSV-1). Methods of antiviral action of propolis involved adding propolis extract at different times during the viral infection cycle. Both propolis extracts exhibited high anti-HSV-1 activity when the viruses were pre-treated with these drugs prior to infection.^31,32^ Anti-HIV-1 activity was observed with propolis samples from several geographic regions. The mechanism of propolis antiviral property in CD4+ lymphocytes appeared to involve, in part, inhibition of viral entry, while propolis had an additive antiviral effect on the reverse transcriptase inhibitor zidovudine.^33^ Isopentyl ferulate in propolis extract has significant inhibitory effects on influenza virus (H3N2) in vitro.^32,34^

### Anticancer property of propolis

The caffeic acid-phenethyl ester (CAPE) in propolis is a potential supportive therapy for patients with oral squamous cell carcinoma (OSCC). CAPE treatment inhibits the proliferation and colony formation and suppresses the cells of OSCC.^35,36^ Furthermore, patients receiving chemotherapy benefit from co-treatment with CAPE. Evidence advocates that CAPE subdues and inhibits cancer lining cells of breast cancer, prostate, lung cancer and oral cancers. CAPE has an inhibitory effect and can be used as a chemical agent to prevent cancer metastasis.35 Treatment with CAPE has shown to defend or guard the vital tissues and organs against the toxins produced during chemotherapy.^35,36^

Propolis has shown overwhelming results and improved quality of life in patients with mucositis, a side effect of radiotherapy and chemotherapy. The natural ingredient was found to be safe and has a characteristic of both prevention and treatment in patients undergoing radiotherapy and chemotherapy.^38^ Table 1 demonstrates evidenced-based properties of propolis‏.

**Table 1:** Key therapeutic properties of propolis for potential bio-dental applications

**Property**	**References**
**Antibacterial**	‏21-23
**Antifungal**	‏25,27-29
**Antiviral**	‏31-34
**Anti-cancer**	‏35,36
**Anti-inflammatory**	‏19,37

### Anti-inflammatory property and propolis

The major component of propolis is CAPE which is a biologically active compound. CAPE has both anti-inflammatory and anti-oxidative properties.^39^ Since CAPE is lipophilic it can easily enter the cell to inhibit the LOX and COX enzymes, which indirectly inhibit arachidonic pathway. The inhibition of arachidonic acid prevents the release of prostaglandins and leukotrienes responsible for inflammation and pain.^40^ CAPE also enhances the production of anti-inflammatory cytokines IL4 and IL10. Furthermore, it decreases infiltration of monocytes and neutrophils.^40,41^

### The role of propolis in dental care

**Figure 4:**
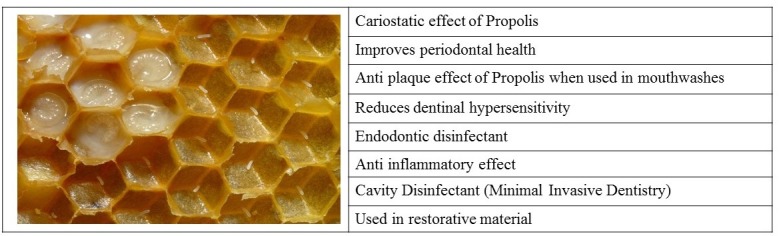
The honeycomb as a major source of propolis extraction and its potential applications in the field of dentistry‏.

Propolis is a natural material mainly obtained from the honeycomb (Figure 4) and has shown promising potential for various bio-dental applications‏.

### Propolis and dental caries

Dental caries is considered as one of the major and chronic dental public health problems. Tailored brushing techniques, diet alteration and use of fluorides play a considerable role in the prevention of carious lesions.^42,43^ Data suggest that the use of "miswak" along with a proper technique as an adjunct to tooth brushing is good for oral as well systemic health.6 Similarly, evidence from different studies assessed the effect of propolis on Streptococcus mutans vulnerability, caries development and glycosyl transferase activity on rats and found that the extract of propolis has cariostatic effects.^44^ Similarly, undisputed results from authors showed that propolis extracts limit plaque formation on the tooth surface, which indirectly reduces dental caries.^45-48^ Furthermore, Durate et al^49^ reported that fatty acids in propolis provide a cariostatic effect by decreasing the tolerance of microorganisms to low pH and slowing down acid production.^50^ Recently, Nam et al^51^ reported that Brazilian propolis possesses significant antimicrobial effects against Steptococcus mutans in the oral cavity by inhibiting the enzyme activity and cell division. He further concluded that propolis could be used as an alternative and natural therapy against the infectious condition of the oral cavity with no reported side effects.^51,52^ A study by Cordoso et al^47^ agrees with the findings of Nam et al,^51^ indicating that ethanolic extract of propolis has no inhibitory action on demineralization of caries process‏.

### Propolis and periodontal health

Multiple and diverse effects of propolis on oral health have led to its use in periodontal diseases. Subgingival irrigation with propolis extracts during periodontal treatment yielded better results than root planing and scaling.^53,54^ Furthermore, propolis extracts when used in gingival pockets, are beneficial for periodontal diseases.^54,55^ A study on the histological and morphological picture established that application of propolis systematically prevents further bone loss in periodontal conditions in rats.^56^ In addition, Gebara et al^57^ reported that in vitro use of propolis extracts not only had antimicrobial activity against periodontopathic bacteria (Capnocytophagagingivalis, Prevotella intermedia, Fusobacterium neucleatum, Porphyromonas gingivalis) but also against microorganisms that cause supra-infection (Staphylococcus aureus, Escherichia coli, and Candida albicans).^57^

### Propolis mouthwashes and toothpastes

Mouthwashes are used as commercial antiseptics and used as a home remedy for better oral hygiene. These mouthrinses can be both cosmetic and therapeutic. Therapeutic mouthwashes reduce bacterial counts, have antiplaque effects, work as an astringent and help in reducing gingivitis and carious lesions.^58-60^ A study assessed the effect of propolis mouthwashes by comparing plaque and gingival index scores at baseline and at a five-day interval. Chlorohexidine mouthwashes were more effective when compared to propolis extract-based mouthwashes.^61^ Furthermore, the effect of propolis mouthwashes on gingival fibroblasts showed less cytotoxicity than chlorohexidine mouthwashes. Ozan et al and Arsalan et al concluded that propolis mouthwashes were not as effective as chlorohexidine mouthwashes in caries prevention.^62,63^ A recent in vitro study by Akca et al showed that ethanolic extract of propolis was more effective against gram-positive bacteria than against gram-negative bacteria in their planktonic state and can be used as an alternative to chlorohexidine in order to avoid its side effects. Studies are required to find the effects of propolis on biofilms.^58^ Research has proven that mouthrinses containing propolis in an alcohol aqueous solution heals intra-buccal surgical wounds; therefore, it plays a role in epithelial repair after tooth extraction and exerts anti-inflammatory effect on orofacial pain.^64^ Propolis in toothpaste was seen to greatly improve oral health and showed inhibitory effect on dental plaque formation, which is considered as the main etiology of most oral diseases. Propolis-based toothpastes should be used as adjuncts to other substances in subjects who are at a higher risk for periodontal-related problem.^65^

### Effect of propolis on dentin hypersensitivity

Dentin hypersensitivity is defined as a sudden sharp short pain arising from tactile, osmotic, thermal or other stimuli from exposed dentin.^66^ There are various theories for dental hypersensitivity. Amongst these theories, the hydrodynamic theory is considered as the most acceptable and relevant. It is proposed that propolis reduces dentinal hypersensitivity by decreasing hydraulic conductance of dentin.^67,68^ A recent study by Hussain et al showed that propolis, when used in the treatment of dentinal hypersensitivity at chair side after bleaching, yielded convincing results.^68^ Similarly, another study by Hongal et al showed contrasting results when Indian propolis was compared with RecaldentTM. RecaldentTM showed significant results in reducing dentinal hypersensitivity.^69^ Similarly, when 5% propolis extract was compared to potassium nitrate in reducing dentinal hypersensitivity no difference was observed between the two groups. Propolis used as a natural desensitizer is still a vague concept and needs further verification through research.^70^

### Propolis used as a cavity disinfectant in vivo

Good caries prognosis is directly related to removal of infected dentin. Due to improved understanding of the caries process, there is a dramatic advancement in the management of carious lesions. Cavity disinfection is an adjunctive method to minimize or reduce bacterial counts in the residual dentin after cavity preparation.^71^ Propolis along with other cavity disinfectants, i.e. APF (acidulated phosphate fluoride) gels, diode lasers and 2% chlorhexidine, was used against S. mutans and L. bacilli and it was observed that there was a significant decrease in bacterial counts in all the groups. Nevertheless, APF gels showed the least reduction, whereas both Brazilian propolis and diode lasers were equally effective when compared to the control group of 2% chlorohexidine.^72^ A randomized controlled trial by Prabhakar et al and evidence from others demonstrated that after minimal invasive hand excavation both aloe vera and propolis can be used as a potential cavity disinfectant.^73-75^

### Effect of propolis against endodontic pathogens

Endodontic infection is the infection of the dental root canal system and the chief etiologic agent of apical periodontitis. The evidence clearly recommends that microorganisms are crucial for the advancement and continuation of diverse forms of apical periodontitis.^76^ The rationale behind the endodontic treatment is to eliminate the infection and to prevent microorganisms from infecting or re-infecting the periradicular tissues.^77^ Ethanol-based propolis was tested as an endodontic disinfectant compared to the conventional disinfectant (chlorohexidine and calcium hydroxide) against gram-positive facultative anaerobe Enterococcus raecalis (E. faecalis) in vitro.^78^ The results showed that antimicrobial effect of propolis was found to be between chlorohexidine and calcium hydroxide. Chlorohexidine was the most effective endodontic antiseptic against E. faecalis. Propolis samples exhibited antimicrobial effects but their efficiency was not beyond chlorohexidine. For propolis to be used as an endodontic irrigant, more human trials are needed to find out cytotoxicity and tissue response of the material.^78,79^ Similarly, Ferreira et al reported the effect of propolis against different endodontic pathogens, concluding that Brazilian propolis was effective against all strains. E. faecalis was considered as the least susceptible strain.^80^

### Propolis and pulp inflammation

An in vitro and in vivo study revealed that propolis has a strong anti-inflammatory effect and can be used as a pulp capping agent. Flavonoids and caffeic acid are the main ingredients in propolis, responsible for anti-inflammatory response by inhibiting the lipoxygenase and arachidonic pathway.^81,82^ In addition, the flavonoids and caffeic acid provide acceleration of the immune system by enhancing the phagocytic activities.^8,81^ There are numerous studies over the years that have demonstrated the anti-inflammatory effects of propolis.^55,81,83,84^ Bachiega et al showed that cinnamic acid and coumaric acid in propolis impede IL-6 and IL-10 but encourage IL-B production by macrophages.^85^ Evidence suggests this anti-inflammatory effect of propolis depends upon the potential dose and route of administration‏.

### Propolis and tooth restorative material

Glass-ionomer cement (GIC) is a fluoride releasing material used for restorative purposes.86-88 GIC is considered as the only material of choice for atraumatic restorative treatment (ART). Favorable characteristics of this material may include biocompatibility, chemical bonding, constant fluoride release, inhibition of bacterial acid metabolism and bactericidal potential.^89^

Propolis, when added to GIC, has a distinct antibacterial and anti-biofilm efficacy and can be used as a promising material in future restoration.^90^ In vitro extracts of propolis were added to GIC for evaluation of microhardness and microleakage. The results showed that GIC treated with propolis resulted in an increase in microhardness with no changes or effects on microleakage.^89^ In addition, when 1% ethanolic extracts of propolis were added to GIC it enhanced the fluoride releasing capacity of GIC without a change in shear bond strength.^91^ Alternatively, a recent study by Subramaniam et al^92^ suggests that physiochemical properties of GIC tend to wear off when propolis is added. In the limelight of the above evidence, it is suggested that GIC with propolis is still a debatable issue and more features of GIC with propolis need to be tested before conclusions regarding their effectiveness can be drawn‏.

### Harmful effects of propolis and future challenges

The most common and reported side effect of propolis is allergy to the resinous wax-cum material. Thirty-seven German beekeepers out of 1051 were allergic to propolis and showed symptoms of skin rashes after working in bee farms professionally.^93,94^ Similarly, Brailo et al reported a subjective case of a 20-year-old women who experienced irregular erosions covered by pseudomembranes involving the lips and oral mucosa. She used propolis-based ointments for treatment of aphthous ulcers.^94^ Moreover, Zirwas and Otto claimed that with time allergic cases of propolis have increased from 0.4% to 1.4%.^95^ Furthermore, due to certain impurities in propolis, there is limited literature to recommend it in pregnant women.^96^ Propolis preparation may contain high levels of alcohol and may result in nausea when taken as an adjunct to metronidazole.^97^ Contents in propolis may interact with antiviral, anticancer, antibiotic and anti-inflammatory drugs and may manifest allergic reactions which may range from eczema, cheilitis, oral pain, labial edema and peeling of lips.^97,98^ Additionally, more research should be carried out to define the parameters of the use of propolis both in the dental and medicinal fields‏.

## Conclusions

Propolis is rated among few natural remedies, which has still maintained its popularity over time due to its wide range of applications in both dentistry and medicine. Its extensive and wide-ranging variety of properties such as anti-inflammatory, anti-bacterial, antiviral and anti-fungal has maintained the focus and attention of many researchers. Most of the work on propolis is in vitro or animal studies. There is a need for human clinical trials to get the best benefit out of this natural ingredient. There is a great need for outlining the algorithms of its use in the dental and medical fields based on its biological properties‏.

## Competing interests

The authors declare that they have no competing interests with regards to authorship or publication of this paper. ‏
